# Development of Chitosan/Sodium Carboxymethylcellulose Complexes to Improve the Simvastatin Release Rate: Polymer/Polymer and Drug/Polymer Interactions’ Effects on Kinetic Models

**DOI:** 10.3390/polym15204184

**Published:** 2023-10-22

**Authors:** Celia López-Manzanara Pérez, Norma Sofía Torres-Pabón, Almudena Laguna, Guillermo Torrado, Paloma M. de la Torre-Iglesias, Santiago Torrado-Santiago, Carlos Torrado-Salmerón

**Affiliations:** 1 Department of Pharmaceutics and Food Technology, Faculty of Pharmacy, Complutense University of Madrid, Plaza Ramón y Cajal s/n, 28040 Madrid, Spain; celiadlo@ucm.es (C.L.-M.P.); almulagu@ucm.es (A.L.); pmtorre@ucm.es (P.M.d.l.T.-I.); 2 Department of Biomedical Science, Faculty of Pharmacy, University of Alcalá de Henares, Ctra Madrid-Barcelona Km 33600, 28805 Madrid, Spain; sofia.torres@uah.es (N.S.T.-P.); guillermo.torrado@uah.es (G.T.); 3 Instituto Universitario de Farmacia Industrial (IUFI), Complutense University of Madrid, Plaza Ramón y Cajal s/n, 28040 Madrid, Spain

**Keywords:** simvastatin, chitosan, carboxymethylcellulose, polyelectrolyte complexes, ionic interaction

## Abstract

Simvastatin (SIM) is a potent lipid-lowering drug used to control hyper-cholesterolemia and prevent cardiovascular diseases. SIM presents low oral bioavailability (5%) because of its low aqueous solubility. In this work, polyelectrolyte complexes (PEC) are developed with different chitosan (CS) and carboxymethylcellulose (CMC) ratios that will allow for an increase in the SIM dissolution rate (2.54-fold) in simulated intestinal medium (pH 4.5). Scanning Electron Microscopy (SEM) images revealed highly porous structures. The changes between both complexes, PEC-SIM:CS:CMC (1:1:2) and (1:2:1), were related to the relaxation of the polymer chains upon absorption of the dissolution medium. Fourier-transform infrared spectroscopy (FTIR), differential scanning calorimetry (DSC) and powder X-ray diffraction (XRPD) studies were used to evaluate the polymer/polymer and drug/polymer interactions on the different PEC-SIM:CS:CMC ratios. In addition, the PEC-SIM:CS:CMC (1:2:1) complex exhibited a high ratio of protonated amino groups (NH_3_^+^) and an increase in intramolecular hydrogen bonds, which were correlated with a high expansion of the interpolymer chains and an increase in the SIM dissolution rate. Different kinetic models such as zero-order, first-order, Higuchi and Korsmeyer–Peppas were studied to evaluate the influence of CS/CMC ionic interactions on the ability to improve the release rate of poorly soluble drugs.

## 1. Introduction

In recent years, the development of chitosan/polyelectrolyte anionic complexes (PECs) has attracted growing interest for the control and modulation of drug release. The electrostatic attractions between the ionized amino group from chitosan (NH_3_^+^) and the carboxylic groups (COO^−^) from anionic polymers are the main interactions for the formation of polyelectrolyte complexes and are widely used for the drug’s controlled release [1,2,3].

Simvastatin (SIM) is an antihyperlipidemic agent, generally used for the treatment of hyperlipidemia. SIM was categorized as class II in the biopharmaceutical classification system, with low solubility in water (0.26 mg/L). SIM showed a sustained release in the gastric environment (SGF pH 2.0) and a faster release in the intestinal environment (SIF pH 6.8) [4]. These different dissolution degrees are related to the lactone ring of SIM, which presents a hydrolyzed process under acidic or alkaline conditions [5]. Solid dispersions with different hydrophilic carriers have proven to be a good technological means to improve the dissolution profiles of poorly soluble drugs [6,7]. Recent studies showed that chitosan (CS) polyelectrolyte complexes at pH close to its pKa (4.6) or higher exhibit a large number of intramolecular protons (H^+^), which causes an increased water uptake and an improved poorly soluble drug dissolution rate [3,8].

Hyperlipidemia is an important risk factor for coronary artery disease (CAD). Recently, SIM has been used in the first line of treatment to prevent different CAD disorders [9,10]. Several studies have shown an association between CAD and nonalcoholic fatty liver disease (NAFLD) [10]. Different previous studies indicated that SIM treatments in animals were associated with hepatotoxicity, myopathy, and histological changes in the quadricep muscles [4]. In the treatment of dyslipidemia in rats, rapid dissolution systems of poorly soluble drugs such as ezetimibe or SIM at low doses showed efficacy results similar to those obtained with higher doses of poorly soluble raw materials [4,9]. Different subchronic toxicities studies on CS/Alginate and CS/CMC complexes were considered practically nontoxic [3,11]. The improvement of SIM dissolution profiles by using CS/CMC complexes with lower drug doses could be suitable to reduce hepatotoxicity. 

CS/carboxymethylcellulose (CMC) complexes have been shown to be suitable for increasing the dissolution profile of different poorly soluble statins [2] and have been used to improve the treatment of different liver disorders [3,12]. These polyelectrolyte complexes based on CS/anionic polymers are pharmaceutical systems that are easy to prepare on an industrial scale [2,8], while the preparation of nanoparticles, nanofibers and lipid nanoparticles [3,4,5] presents greater difficulties [3,4,5].

CS polyelectrolyte complexes with different natural anionic polymers such as xanthan, sodium carboxymethylcellulose or alginate exhibit different ionic interactions within the polyelectrolyte network (Table 1) [8,11,13]. The different ratios of CS within the ionic complex modify its ionic interactions and produce different release rates at pH 2.0, 4.5, 6.8 and 7.4 [1,3,14].

A diverse behavior of the polyelectrolyte complexes (PECs) was observed in different gastrointestinal environments (Table 1). Low dissolution profiles were reported at pH < 4.5 [1,14,15]. At these pH values, the CS chains are protonated (NH_3_^+^) and the CMC chains (pKa around 3.5) exhibit an anionic charge (COO^−^) and form a slowly dispersible network structure [16,17]. In this acidic medium, most of the CS chains were used in CS/CMC polyelectrolyte interactions, leaving few free cationic groups (NH_3_^+^), which were related to low ionic repulsions between the CS cationic groups within the molecular structure [15]. However, at pH 4.5, the CS/anionic polymer complexes showed higher dissolution profiles with different kinetics [1,3,14]. The presence of high CS proportions indicated a high number of free cationic groups (NH_3_^+^) within the complex, increasing the separation of interpolymeric chains and intramolecular hydrogen bonds [2,15]. The high repulsion between the protonated chains (NH_3_^+^) of the CS chains and the intramolecular hydrogen bonds favored wettability and improved the drug dissolution rate [18]. Finally, the CS/anionic complexes in alkaline media (at pH 6.8 and 7.4) showed a non-protonated network, which was related to fast dissolution rates [8,12,13]. 

The different polymer/polymer and drug/polymer interactions observed in Table 1 indicate good fits to first-order or zero-order kinetics in the pH range between 4.5 and 7.4 [2,3]. In addition, anionic drugs, such as SIM, have a higher interaction degree with the ratio CS:CMC (1:1) compared to CS:CMC (1:9), as the interaction decreases with higher CMC proportions, and this has been attributed to a higher presence of free negative charges on CMC [16].

The objective of this work was to study the increase in the SIM dissolution rate at pH 4.5 from polyelectrolyte complexes with different CS:CMC ratios. Characterization techniques like scanning electron microscopy (SEM), Fourier-transform infrared spectroscopy (FTIR), powder X-ray diffraction (XRPD) and differential scanning calorimetry (DSC) will be used to study several CS/CMC and drug/polymer interactions within the different PEC-SIM:CS:CMC complexes. The release of SIM and its kinetic studies at pH 4.5 also will be used to select the best ratio of CS within the CS/CMC complex for improving the drug dissolution rate. 

## 2. Materials and Methods 

### 2.1. Materials

Chitosan (CS) with a high molecular weight (Mw 310–375 kDa, and a deacetylation degree > 75%) and Carboxymethyl cellulose sodium salt (CMC) with high viscosity (Mw 1500–2500 Da and a substitution degree of 70%, equivalent to 0.7 carboxymethyl groups per anhydroglucose unit) were purchased from Sigma-Aldrich (Madrid, Spain). Simvastatin (SIM) was provided by Normon (Madrid, Spain). The water used in these studies was obtained from a Milli-Q water purification system (Billerica, MA, USA). All other chemicals were at least of pharmaceutical grade.

### 2.2. Methods

#### 2.2.1. Preparation of Formulations

Simvastatin raw material (SIM-RM) was used as the reference for characterization (SEM, XPRD, and DSC) and dissolution studies. The physical mixture PM-SIM:CS:CMC (1:1:2) was prepared by mixing 100 mg of SIM with 100 mg of CS and 200 mg of CMC in a ceramic bowl. Polyelectrolyte complexes were prepared containing different CS/CMC ratios: PEC-SIM:CS:CMC (1:1:1), PEC-SIM:CS:CMC (1:1:2), and PEC-SIM:CS:CMC (1: 2:1) (*w*/*w*). For the preparation of the different polyelectrolyte complexes a CS gel was made by swelling 100 mg of CS in 2 mL of 10% acetic acid aqueous solution over 15 min in a ceramic bowl. Then, 100 mg of CMC was mixed for 2 h in a ceramic bowl.

The pH of hydrogel network was adjusted to 4.5 with a NaOH solution (1.25 mol/L). A SIM dissolution was prepared by adding 100 mg of SIM in 500 µL of ethanol in a vortex (Fisherbrand^TM^; Milan, Italy) at 2500 rpm for 2 min. The SIM solution was incorporated into the CS/CMC gel and mixed for 15 min in a ceramic bowl. The formulation was dried at 40 °C for 24 h and sieved between 0.850 and 0.297 mm.

#### 2.2.2. Scanning Electron Microscopy (SEM) Studies

Samples were mounted on a double-faced adhesive tape and sputtered with a thin gold–palladium layer using a sputter coater Emitech K550X (Quorum Technologies; Lewes, UK). After coating, the hydrogel samples were analyzed with a Jeol JSM-6400 scanning electron microscope (Jeol Ltd., Peabody, MA, USA). All micrographs were the product of secondary electron imaging used for surface morphology identification at an accelerating voltage of 20 kV and a magnification of 800×.

#### 2.2.3. In Vitro Drug Release

Drug release studies were performed with SIM raw material (SIM-RM), physical mixture PM-SIM:CS:CMC (1:1:2) and polyelectrolyte complexes PEC-SIM:CS:CMC (1:1:1), PEC-SIM:CS:CMC (1:1:2) and PEC-SIM:CS:CMC (1:2:1).

These studies were performed using the United States Pharmacopeia (USP) paddle method (apparatus 2) in Erweka DT 80 (Erweka GmbH; Langen, Germany) dissolution equipment with a rotational speed of 50 rpm, at a temperature of 37.0 ± 0.5 °C, and 500 mL of dissolution medium (0.05 M sodium acetate buffer) adjusted to pH 4.5 (USP44-NF39, 2021).

A sample of 5 mL was withdrawn and filtered through a 0.45 m filter (Acrodisc^®^ HPVL 0.45 m) at 5, 15, 30, 45, 60, 90, 120, 150, 180, 210, 240, 300, 360, 420 and 480 min. The quantity of SIM was determined at 238 nm using a UV-VIS spectrophotometer (Jasco^®^ Analitica S.L.; Madrid, Spain). The cumulative amount of SIM released from the system was determined from the following calibration curve, y = 0.0604 × (µg/mL) − 0.0143 (*r^2^* = 0.9950) over the range of 0.25–15 µg/mL, with a limit of detection of 0.1 µg/mL and a limit of quantification of 0.25 µg/mL. This method was validated according to ICH Q2 (R1) (CPMP/ICH/381/95). Each determination at each time was performed in triplicate and the error bars on the graphs represent the standard deviation. Finally, the kinetics parameters from the zero-order release kinetics, the first-order kinetics model, the Higuchi model, and the Korsmeyer–Peppas model were calculated with the DDSolver 1.0 software [19,20].

#### 2.2.4. Fourier-Transform Infrared Spectroscopy (FTIR)

The different samples were prepared by weighing different amounts of the different complexes (equivalent to 40 mg of SIM) and mixed with 800 mg of potassium bromide. The different samples were subjected to pressure at 10 T (Carver hydraulic press Model C-3912 (Wabash, IN, USA). The analysis was made using a Fourier-transform infrared spectroscopy (FTIR) Perkin Elmer 1600 FTIR spectrophotometer (Perkin Elmer, Inc., MA, USA). The spectra were obtained at a 2 cm^−1^ resolution with an average of 64 scans. The infrared region was analyzed in the range of 400–4000 cm^−1^.

#### 2.2.5. Differential Scanning Calorimetry (DSC)

DSC scans for the different formulations were obtained using an automated thermal analyzer system (Mettler Toledo TC 15, TA controller). Temperature calibration was performed using Indium Calibration Reference Standard (transition point 156.60 °C). All dried samples were accurately weighed into aluminum pans and hermetically sealed with aluminum lids and heated from 0 to 250 °C at a rate of 10 °C/min under constant purging of dry nitrogen at 30 mL/min. An empty pan was sealed and used as a reference with the same sample conditions.

#### 2.2.6. X-ray Powder Diffractometry (XRPD)

The XRPD studies for the different formulations were performed on a Philips X’Pert-MPD X-ray diffractometer (Malvern Panalytical; Almelo, The Netherlands) at the CAI (Centro de Asistencia a la Investigación, Complutense University of Madrid, Spain). The samples were radiated using a monochromatized CuKα radiation (λ = 1.542 Å) and analyzed between the 5 and 40° (2θ) range at a step size of 0.04° and a time of 1 s per step.

#### 2.2.7. Drug Release Kinetics

The release kinetics were fitted using zero-order release kinetics, the first-order kinetics model, and the Higuchi model [19,20].

(1) $$M_{t}/M_{\infty }=K_{0}\;t\;\mathrm{Zero}-\mathrm{order}\;\mathrm{model}$$

(2) $$M_{t}/M_{\infty }=K_{H}\;t^{0.5}\;\mathrm{Higuchi}\;\mathrm{model}$$

(3) $${Ln\;[100-(M}_{t}/M_{\infty })]=-K_{1}\;t\;\mathrm{First}-\mathrm{order}\;\mathrm{model}$$

where $M_{t}/M_{\infty }$ is the fractional drug released at time *t* (min) from the total amount released *K*_0_ (min^−1^)_,_ and *K_H_* (min^−1/2^) and *K*_1_ (min^−1^) are the kinetic dissolution constant for the zero-order, Higuchi, and first-order kinetic models, respectively, which characterize release as a function of time t.

Furthermore, the effects of polyelectrolyte complex formation on the SIM release were analyzed according to the Korsmeyer–Peppas Equation for $M_{t}/M_{\infty }$ < 0.6, which can be expressed as the following Equation [19]:

(4) $$M_{t}/M_{\infty }=k_{d}\;t^{n}\;\mathrm{Korsmeyer}-\mathrm{Peppas}\;\mathrm{Equation}$$

where $M_{t}/M_{\infty }$ is the fractional drug released at time *t* (min), *K_d_* (min^−*n*^) is the kinetic dissolution constant and *n* is a diffusional exponent characteristic of the release as a function of time *t*. In the drug release, the exponent *n* is the diffusional constant that characterizes the drug release transport mechanism. Due to the spherical shape of the polyelectrolyte complex, the drug release was evaluated for 0.43 < *n* < 0.85, as used for spherical shapes. When *n* = 0.43, a Fickian diffusion process was observed; the drug diffuses through the polymeric network, which is the dominant release mechanism. When the *n* values are 0.43 < *n* < 1, an anomalous transport (non-Fickian) drug diffusion occurs. Anomalous diffusion assumed that the mechanism of SIM release was a combination of swelling, erosion and diffusion. When *n* = 1, a Case II transport could be observed. The *n* value of about 1.0 indicates that polymer relaxation, polymer dissolution, or erosion are the dominant mechanisms [19,21]. The relationship of the different mathematical models with the different parameters calculated for the polyelectrolyte complexes is briefly described below.

The high *r^2^* values in the zero-order model indicate that polymer relaxation or erosion is the dominant mechanism and is related to Case II transport for the Korsmeyer–Peppas Equation. Different systems containing CS complexes present a good fit to zero order with a sustained release of >60% at 3 h [3].

The high *r^2^* values in the first-order model indicated swelling and erosion phenomena and could be related to non-Fickian (anomalous transport) for the Korsmeyer–Peppas equation. The CS complexes with good fits to the first-order model will show high percentages of dissolution at initial times [13,14].

The high *r^2^* values in the Higuchi model indicate the better fit of release data for diffusion kinetics, which are related to a Fickian diffusion process in the Korsmeyer–Peppas equation. The CS complexes with high ratios of this polymer will show good fits to the Higuchi model with high dissolution percentages at initial times [8,22].

#### 2.2.8. Statistics Analysis 

Differences between the obtained values (Mean ± SE) for the prepared formulae and the control formulae were explored using a one-way analysis of variance (ANOVA), followed by an appropriate post hoc test in the case of the presence of a significant difference. A *p*-value of less than 0.05 was considered a criterion for a statistically significant difference (Statgraphics^®^ Plus, version 5.1).

## 3. Results

### 3.1. In Vitro Drug Release

**Figure 1:**
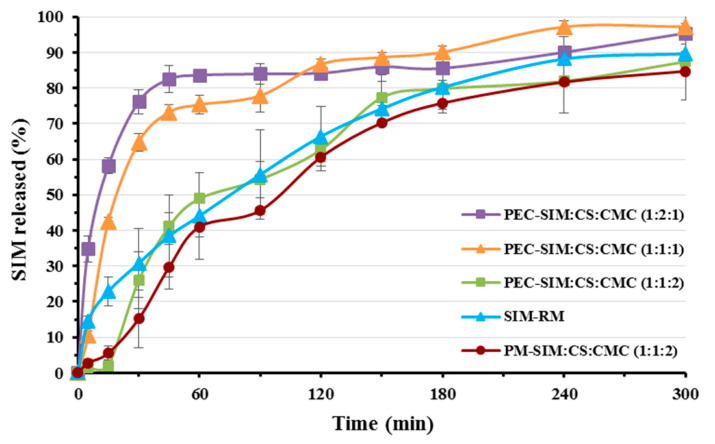
Release profiles at pH 4.5 for SIM-RM, physical mixture PM-SIM:CS:CMC (1:1:2) and the following polyelectrolyte complexes with different ratios of CS/CMC: PEC-SIM:CS:CMC (1:1:1), PEC-SIM:CS:CMC (1:1:2), and PEC-SIM:CS:CMC (1:2:1).

Figure 1 shows the dissolution rate at pH 4.5 for the SIM-RM, physical mixture PM-SIM:CS:CMC (1:1:2) and the polyelectrolyte complexes with different ratios of CS/CMC: PEC-SIM: CS:CMC (1:1:1), PEC-SIM:CS:CMC (1:1:2), and PEC-SIM:CS:CMC (1:2:1).

The dissolution profile of the SIM-RM showed a slow initial release rate at 15 and 60 min (22.92 ± 4.11% and 44.10 ± 12.25%, respectively) and a sustained release rate up to 5 h. This dissolution rate was analogous to those observed for SIM raw material in similar dissolution media [23]. The physical mixture PM-SIM:CS:CMC (1:1:2) presented a significantly delayed drug release (*p* < 0.05) during the first stage of dissolution (5.60 ± 1.87% at 15 min) and a second stage similar to SIM-RM (see Figure 1). In the physical mixture, the absence of ionic interactions between the CS and CMC chains produces a high presence of anionic charge (COO^−^) from CMC, which promotes a hydrogel structure formation in the dissolution medium. This hydrogel is slowly dispersed during this initial stage of the SIM dissolution rate [16,17]. After 15 min, the cationic groups (NH_3_^+^) from the CS favored the repulsion of the polymeric chains within the hydrogel structure [15], displaying SIM dissolution profiles similar to SIM-RM. During this second stage of the physical mixture’s dissolution process, the high hydrophobic character of SIM and its low solubility were the limiting factors for its dissolution rate [6,24]. 

In weak acidic medium (acetate buffer pH 4.5), polymer/polymer ionic interactions from the CS and CMC complexes produced a dense interpenetration network which promoted the SIM molecules’ inclusion inside its structure. The deprotonated carboxylic groups (COO^−^) from CMC produced a greater number of hydrogen bonds with the protonated amino groups (NH_3_^+^) from CS polymeric chains, and interacted with the dissolution medium, favoring the presence of water molecules on the surface of these complexes. Furthermore, chitosan (CS) polyelectrolyte complexes at a pH close to their pKa (4.6) exhibited a large number of intramolecular protons (H^+^), which caused higher water absorption and wettability and therefore a better dissolution rate of the poorly soluble drug (SIM) [3,8].

The polyelectrolyte complex with the lowest CS/CMC ratio, PEC-SIM:CS:CMC (1:1:1), showed a dissolution profile with significant increases (*p* < 0.05) at 15 and 60 min (1.86 and 1.71-fold, respectively) in comparison to SIM-RM. These results indicated that the presence of the low ratios of both hydrophilic polymers CS and CMC significantly (*p* < 0.05) increase the SIM-RM dissolution rate. However, high CMC amounts in polyelectrolyte complexes produced sustained dissolution profiles. Thus, the PEC-SIM:CS:CMC (1:1:2) showed an important decrease in the dissolution rate with values at 15 and 60 min of 26.00 ± 7.96% and 48.94 ± 1.80%, respectively (Figure 1). This complex showed a dense ionized layer with a high ratio of CMC chains, which favored the sustained release profile (Figure 1). Similar delays in dissolution profiles have previously been observed with polyelectrolyte complexes with higher ratios of anionic chains [8]. Finally, the presence of high proportions of CS in the polyelectrolyte complex PEC-SIM:CS:CMC (1:2:1) showed an important burst effect at initial time points (0–30 min) and a rapid dissolution profile with a significant increase (*p* < 0.05) at 15 min and 60 min, respectively (2.54- and 1.90-fold, respectively) in comparison to SIM-RM. This important improvement in dissolution rate was attributed to a high swelling process observed for the high-proportion CS complex during the dissolution studies. The presence of higher proportions of CS for PEC-SIM:CS:CMC (1:2:1) increases the repulsion between the NH_3_^+^ cationic charges of the CS chains, which favors the disentanglement of the network. In addition, the increases in intramolecular hydrogen bonds favored surface wettability of hydrophobic drugs [18]. Similar anomalous behavior has been previously observed in other CS/CMC complexes, which showed increased dissolution rates with high CS proportions within the interpenetration network [17].

### 3.2. Scanning Electron Microscopy (SEM)

**Figure 2:**
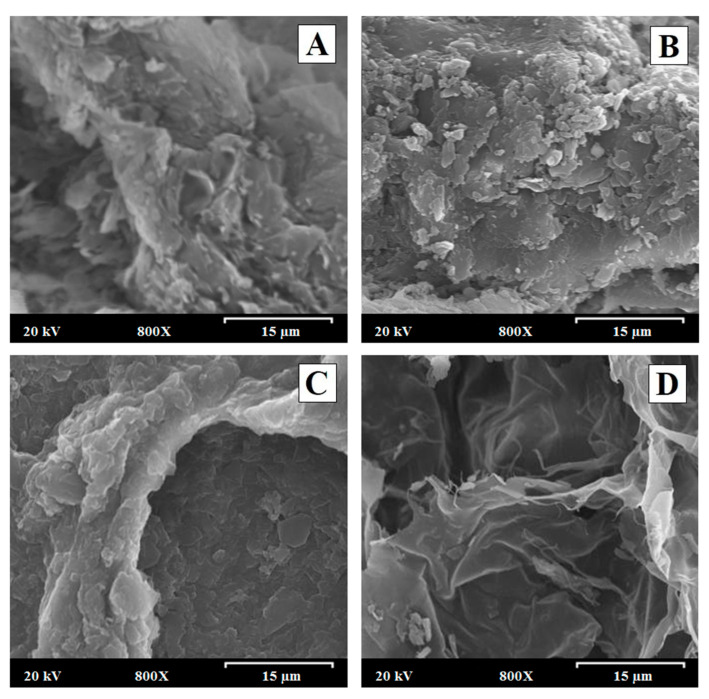
Scanning electron micrographs of (**A**) PEC-SIM:CS:CMC (1:1:2) and (**B**) PEC-SIM:CS:CMC (1:2:1) before the dissolution study. Scanning electron micrographs of the polyelectrolyte complexes (**C**) PEC-SIM:CS:CMC (1:1:2) and (**D**) PEC-SIM:CS:CMC (1:2:1) after 15 min at pH 4.5. Original magnification is 800× and the scale bar is equal to 15 μm.

Figure 2A,B displays scanning electron micrographs of PEC-SIM:CS:CMC (1:1:2) and PEC-SIM:CS:CMC (1:2:1) before the dissolution study. Figure 2D shows the polyelectrolyte complexes PEC-SIM:CS:CMC (1:1:2) and PEC-SIM:CS:CMC (1:2:1) after 15 min at pH 4.5 in order to study the influence of different proportions of CS and CMC within the interpolymer network structure. All micrographs of these formulations were taken at a magnification of 800×.

The polyelectrolyte complex PEC-SIM:CS:CMC (1:1:2) (Figure 2A) showed small crystalline acicular SIM particles with sizes of 0.5–5.0 µm on the surface of both CS and CMC fibers [2]. This microcrystalline morphology is frequent in poorly soluble drugs such as SIM [22,23]. On the other hand, PEC-SIM:CS:CMC (1:2:1) polyelectrolyte complex (Figure 2B) exhibited an aggregated particle structure with a smooth surface characteristic of polyelectrolyte CS complexes [2,16]. A decrease in the number of crystalline particles of SIM was attributed to an entrapment of SIM molecules within the polyelectrolyte complexes during the manufacturing process [2].

After 15 min in the dissolution medium (acetate buffer pH 4.5), the different interpolymeric complexes showed a hydrogel interpolymer structure with interconnected pores which were created during the lyophilization process [25]. The PEC-SIM:CS:CMC (1:1:2) with a higher CMC amount (Figure 2C) displayed a dense interpolymer structure that reduces the presence of connections (channels) with pore sizes of 40–80 µm. Similar dense interpolymeric structures have been previously described with high proportions of anionic polymers in CS/alginate complexes [20]. However, the PEC-SIM:CS:CMC (1:2:1) with a higher CS amount (Figure 2D) revealed a slight interpolymer structure with a large number of channels within the system [22,26]. These differences in the surfaces were related to a high repulsion between the cationic moieties present in the complexes with high proportions of chitosan [20,25].

The increase in polymeric channels with higher ratios of CS observed in Figure 2D reduces the thickness of the interpolymeric structures and improves the swelling process of the polyelectrolyte complexes, increasing the surface wettability of hydrophobic drugs such as SIM and enhancing dissolution rates with high CS proportions.

### 3.3. FTIR Spectroscopy Analysis

**Figure 3:**
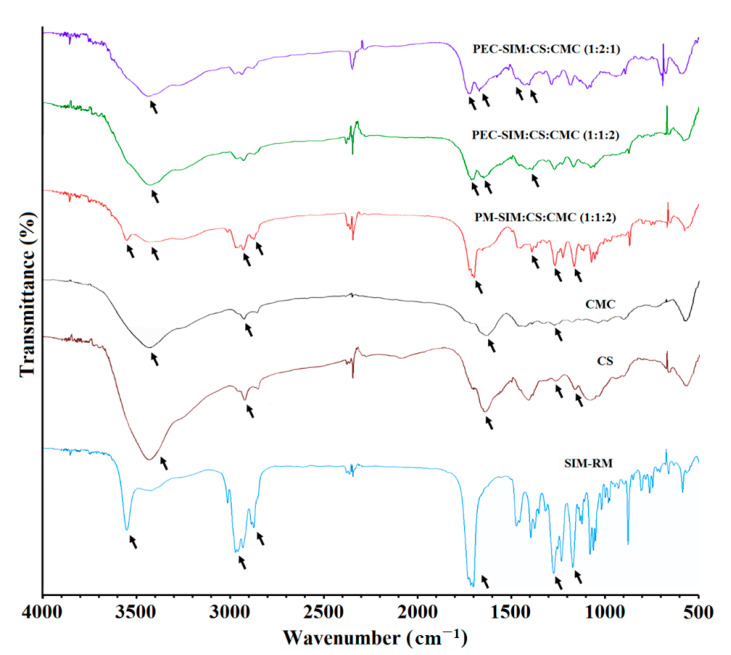
FTIR spectra of SIM-RM, CS and CMC raw materials, physical mixture PM-SIM:CS:CMC (1:1:2) and polyelectrolyte complexes PEC-SIM:CS:CMC (1:1:2) and PEC-SIM:CS:CMC (1:2:1).

Figure 3 showed the FTIR characterization of SIM, CS, and CMC raw materials, physical mixture PM-SIM:CS:CMC (1:1:2) and polyelectrolyte complexes PEC-SIM:CS:CMC (1:1:2) and PEC-SIM:CS:CMC (1:2:1). The spectrum of SIM raw material showed the following characteristic bands: at 3551 cm^−1^, corresponding to alcoholic O–H stretch vibrations; both bands at 2970 and 2831 cm^−1^, corresponding to asymmetric and symmetric C–H stretching vibrations; the band at 1698 cm^−1^, attributed to carbonyl groups (–C=O); and the bands at 1268 cm^−1^ and 1166 cm^−1^, corresponding to lactone groups (–C–O) [5,7,26]. The CS polymer exhibited a broad band at 3440 cm^−1^, corresponding to both O–H and N–H stretching vibrations; a band at 2926 cm^−1^, attributed to C–H bands; a band at 1641 cm^−1^, related to carbonyl groups –C=O from N–H amide I group; and bands at 1263 and 1159 cm^−1^ (groups –C–O) [5,21]. The CMC spectrum showed a band at 3433 cm^−1^, corresponding to O–H stretching vibrations, while the band at 2923 cm^−1^ is due to C–H stretching vibrations of the –CH_2_ groups; the bands at 1625 and 1263 cm^−1^ correspond to the stretching vibrations of the both –C=O carbonyl groups and –C–O groups [27].

The presence of SIM in the physical mixture PM-SIM:CS:CMC (1:2:1) was observed in Figure 3 with a characteristic band at 3552 cm^−1^, attributed to the O–H stretching vibration from hydroxyl groups; a broad band around 2952 and 2830 cm^−1^, corresponding to the asymmetric and symmetrical vibrations for C–H bonds; both bands at 1698 and 1392 cm^−1^, which were characteristic to lactone and carbonyl groups –C=O; and bands at 1267 and 1166 cm^−1^, corresponding to the stretching vibrations of–C–O bond for the lactone group. No changes were observed in the characteristic stretching vibrations bands for CS and CMC polymers. The foregoing spectroscopic data suggest that there is no drug/polymer or polymer/polymer incompatibility during the elaboration process of the physical mixtures [28].

The spectra of the polyelectrolyte complexes PEC-SIM:CS:CMC (1:1:2) and PEC-SIM:CS:CMC (1:2:1) exhibited considerable changes. The absence of the vibration band at 3552 cm^−1^ was observed for both polyelectrolyte complexes and was attributed to the inclusion of SIM within the polyelectrolyte complex [21]. However, these complexes exhibited a higher intensity for the band at 3423 cm^−1^, corresponding to the O–H and N–H stretching vibration from the functional groups present in CS and CMC. These bands were attributed to the presence of intramolecular hydrogen bonds and indicate mild interactions between the drug and chitosan [5]. In addition, PEC-SIM:CS:CMC (1:1:2) and PEC-SIM:CS:CMC (1:2:1) showed significant changes for the bands at 1710 and 1646 cm^−1^ corresponding to carboxylic groups of SIM and CMC and the carbonyl groups (–C=O) of the amide I group of CS. The characteristic band at 1497 cm^−1^ was attributed to a high interaction between the protonated amine groups on CS and the anionic carboxyl groups on CMC during CS/CMC complexation [17,29]. Furthermore, PEC-SIM:CS:CMC (1:2:1) exhibited a slight shift in the vibration band at 1655 cm^−1^, which could be related to the presence of free protonated amino groups (NH_3_^+^), which correlate with a significant expansion of the interpolymeric chains [17] and maintain the vibration band at 1497 cm^−1^, characteristic of an ionic interaction during CS/CMC complexation [17,29].

### 3.4. DSC Studies

**Figure 4:**
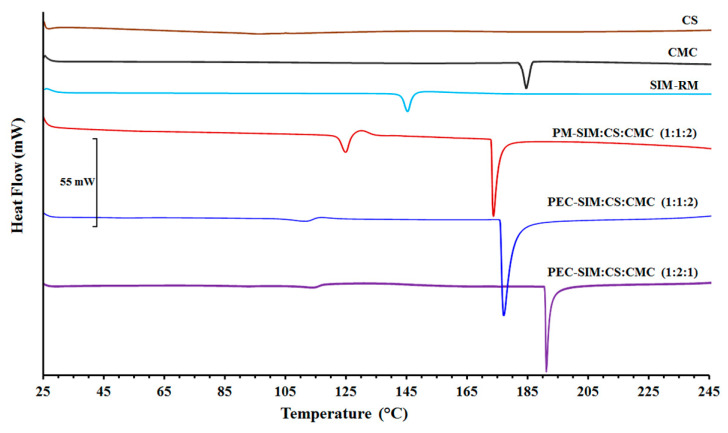
DSC thermograms of SIM-RM, CS and CMC raw materials, physical mixture PM-SIM:CS:CMC (1:1:2) and both polyelectrolyte complexes PEC-SIM:CS:CMC (1:1:2) and PEC-SIM:CS:CMC (1:2:1).

Figure 4 shows the DSC scans for SIM, CS, CMC raw materials, physical mixture PM-SIM:CS:CMC (1:1:2), and both polyelectrolyte complexes PEC-SIM:CS:CMC (1:1:2) and PEC-SIM:CS:CMC (1:2:1).

The SIM scan was characterized by an endothermic peak at 146.77 °C with an endothermic value of −54.63 J/g, typical of an orthorhombic microcrystalline form [6,30]. However, CS showed the absence of an endothermic peak, which is characteristic of an amorphous substance [8], while CMC exhibited a broad endothermic peak at 184.24 °C, attributed to a semicrystalline substance [31]. The glass transition temperature (Tg) for CS was observed between 115 and 120 °C [22,27]. However, the Tg of CMC was not observed in the DSC curve due to a high presence of intermolecular bonds between the cellulose chains, which made its determination difficult [31]. The physical mixture PM-SIM:CS:CMC (1:1:2) exhibited a slight displacement of the endothermic peak at 124.97 °C for SIM and a second endothermic peak at 174.10 °C for CMC. The shift at low temperatures of both SIM and CMC could be due to displacements induced by the loss of water trapped in the CS during DSC exploration [15].

The displacement between both endothermic peaks indicated good compatibility among the SIM and CS/CMC polymer chains [7,14].

Both thermograms of PEC-SIM:CS:CMC (1:1:2) and PEC-SIM:CS:CMC (1:2:1) showed a first event at 112.51 and 115.92 °C, respectively. These results were attributed to the loss of water entrapped in the CS during the DSC exploration [14,15,17]. The absence of the characteristic SIM peak in both interpolymeric complexes indicated an entrapment of the SIM within the CS/CMC network [11,30]. The presence of high endothermic peaks at similar temperatures for PEC-SIM:CS:CMC (1:1:2) and PM-SIM:CS:CMC (1:1:2) indicated that the inclusion of SIM within the complex did not produce changes inside the CS/CMC interpolymeric network [14,16]. However, the shift at higher temperatures (191.92 °C) for PEC-SIM:CS:CMC (1:2:1) was related to interactions between the carboxyl groups from the CMC polymer and the amino groups from CS polymer, and due to a high mobility of the protonated amino groups (NH_3_^+^) from cationic chains of CS [14]. This result is consistent with the different ionic interactions observed in the FTIR studies.

### 3.5. X-ray Powder Diffractometry (XRPD)

**Figure 5:**
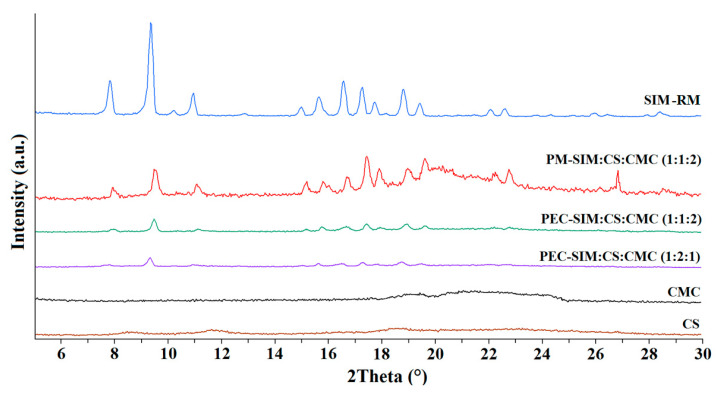
XRPD of SIM-RM, CS and CMC raw materials, the physical mixture PM-SIM:CS:CMC (1:1:2) and the polyelectrolyte complexes PEC-SIM:CS:CMC (1:1:2) and PEC-SIM:CS:CMC (1:2:1).

In Figure 5, the X-ray diffraction patterns of SIM, CS and CMC raw materials, the physical mixture PM-SIM:CS:CMC (1:1:2) and the both polyelectrolyte complexes PEC-SIM:CS:CMC (1:1:2) and PEC-SIM:CS:CMC (1:2:1) are shown. SIM-RM showed a crystalline structure with representative peaks at 8.14, 9.22, 11.09, 16.71, 17.22 and 18.80 °2θ [4,6,7].

The CS swelling process in acetic acid solution and the subsequent drying process produced decreases in the semi-crystalline regions that were attributed to the interactions between the hydroxyl and amino groups of the CS [8,21]. The CMC showed a semicrystalline halo between 18 and 26.5 °2θ, characteristic of cellulosic polymers [26]. The physical mixture PM-SIM:CS:CMC (1:1:2) showed the characteristic peaks of the SIM-RM and a broad semicrystalline halo between 18.5 and 26.5 °2θ corresponding to the CS and CMC polymers (Figure 5). The decrease in the intensity of SIM peaks in PM-SIM:CS:CMC (1:1:2) was related to a dilution effect [7].

The X-ray spectra of both formulations, PEC-SIM:CS:CMC (1:1:2) and PEC-SIM:CS:CMC (1:2:1), exhibited decreases in CS and CMC semicrystalline halos (8.0–9.8 and 18–26.5 °2θ) due to the formation of CS/CMC complexes [32]. These changes in crystallinity indicate that the electrostatic interactions between CS and CMC described in FTIR spectra resulted in the destruction of the semicrystalline structure of both polymers [11,33,34]. Furthermore, PEC-SIM:CS:CMC (1:2:1) and PEC-SIM:CS:CMC (1:1:2) showed a decrease in intensity for the different SIM diffraction peaks compared to PM-SIM:CS:CMC (1:1:2). This result confirms the inclusion of the SIM within the polyelectrolyte complex, as observed in the SEM, FTIR and DSC studies. In this approach, the interactions between the drug and the CS/CMC complex produce a significant decrease in the SIM crystallinity; therefore, its higher Gibbs free energy resulted in rapid drug dissolution [4].

### 3.6. Kinetic Studies

**Figure 6:**
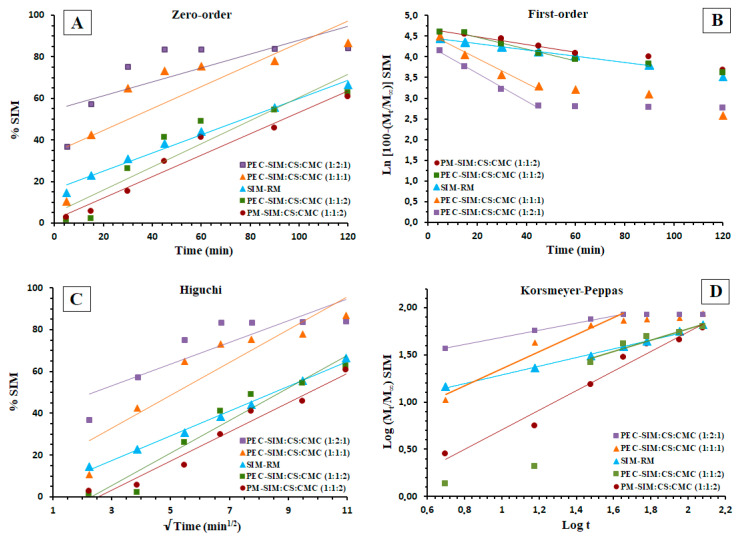
(**A**) Zero-order, (**B**) first-order, (**C**) Higuchi, and (**D**) Korsmeyer–Peppas kinetic models applied for SIM-RM, physical mixture PM-SIM:CS:CMC (1:1:2) and polyelectrolyte complexes PEC-SIM:CS:CMC (1:1:1), PEC-SIM:CS:CMC (1:1:2), and PEC-SIM:CS:CMC (1:2:1).

The drug release kinetics for SIM raw material, physical mixture and the different polyelectrolyte complexes are shown in Table 2 and Table 3 and Figure 6. SIM-RM showed a good fit to the first-order and Higuchi models (*r*^2^ of 0.9980 and 0.9944, respectively) related to a diffusion model. The low value of *K*_1_ (−0.0076 min^−1^) for SIM-RM has been related to the poor solubility of the pure drug alone. In the Korsmeyer–Peppas model (Table 3), the SIM release mechanism (*n* of 0.4597) exhibited anomalous (non-Fickian) transport. Similar values of *n* were previously observed with different poorly soluble drugs [8,30]. However, the PM-SIM:CS:CMC (1:1:2) showed a better fit at zero order (*K*_0_ 0.4361 min^−1^), and, in the Higuchi model, it was possible to observe a slight increase in the *K_H_* value (7.1215 min^−1/2^) compared to SIM-RM. These results could be related to the hydrophilic effect of CS polymeric chains [19,30]. The Korsmeyer–Peppas model showed a value of *n* (1.0395) that was related to a combination of several events: relaxation of the swollen hydrogel and erosion and diffusion mechanisms [8,19,35]. These results could be related to a slow initial swelling process (15 min) which modulates this zero-order kinetics. Furthermore, the polymeric CS chains in these physical mixtures showed an important hydrophilic effect which favors the diffusion kinetics of the SIM [19,30].

The polyelectrolyte complex PEC-SIM:CS:CMC (1:1:1) favors the inclusion of SIM molecules within the complexes formed via the interaction between the cationic polymeric chains (NH_3_^+^) from CS and the anionic polymeric chains (COO^−^) from CMC. The first-order and the Higuchi models showed poor fits to the kinetic models (*r*^2^ of 0.9686) compared to SIM-RM and PM-SIM:CS:CMC (1:1:2), respectively. The Korsmeyer–Peppas model for PEC-SIM:CS:CMC (1:1:1) showed a value of *n* (0.8584), indicating a fit to an anomalous transport. These increases in the *n* values compared to SIM-RM have been related to a rapid swelling and a disintegration of the interpolymeric network structure [19,20]. The polymeric complex PEC-SIM:CS:CMC (1:1:2) showed a delay in the initial drug release followed by a sustained release with good fits to the zero-order (*K*_0_ 1.3013 min^−1^) and Higuchi models (*K_H_* 12.276 min^−1/2^). This remarkable sustained dissolution release was attributed to a high number of anionic chains (COO^−^) from CMC within the polyelectrolyte complex. 

The Korsmeyer–Peppas kinetics exhibited high values of *n* (0.5921), attributed to a dense layer of ions between the cationic and anionic charged groups. In the dissolution medium, the expansion of the CS chains was favored by the repulsion between cationic and anionic charges, which forms a hydrogel layer around the interpolymeric complex [8,35].

The presence of high CS ratios in the polyelectrolyte complexes PEC-SIM:CS:CMC (1:2:1) showed slight improvements for the first-order (*r*^2^ of 0.9755) and Higuchi kinetic models (*r*^2^ of 0.9950). The Korsmeyer–Peppas model showed values of *n* (0.4023) for PC-SIM:CS:CMC (1:2:1) with a better fit to a Fickian diffusion model for these polymeric systems (Figure 6, Table 2 and Table 3) [20]. The low value of *n* in the Korsmeyer–Peppas model could be attributed to a combination of diffusion and relaxation between the NH_3_^+^ groups from CS and the COO^−^ groups from CMC polymeric chains [21]. The presence of the hydrophilic polymer CS within the PEC-SIM:CS:CMC (1:2:1) increased the swelling process and produced a high mobility of the protonated amino groups (NH_3_^+^) from the CS cationic chains, which enabled a rapid initial release rate of the SIM (in a time range of 0–45 min) [19,22]. 

## 4. Conclusions

The elaboration and characterization of different polyelectrolyte complexes, PEC-SIM:CS:CMC (1:1:1), PEC-SIM:CS:CMC (1:1:2) and PEC-SIM:CS:CMC (1:2:1), were carried out. These complexes displayed different CS/CMC interactions that were correlated with different SIM release rates at pH 4.5. SEM studies showed different changes in the surface morphology of the interpolymeric network related to the CS amount within the CS/CMC interpolymeric complexes. These complexes exhibited a high swelling process within the CS/CMC, which was confirmed via FTIR, DSC and XRPD studies. The different polyelectrolyte complexes studied showed different interactions between the protonated amino groups (NH_3_^+^) from the CS chains and the anionic groups (COO^−^) from the CMC chains. In addition, the presence of free amino groups (NH_3_^+^) was shown to be dependent on the CS and CMC ratios within the interpenetration network. High CMC ratios in PEC-SIM:CS:CMC (1:1:2) increase the number of anionic groups (COO^−^), which produces a dense structure with high polymer/polymer interactions, resulting in a more sustained SIM release profile, avoiding side effects such as hepatotoxicity. 

Dissolution studies (at pH 4.5) indicated that high CS proportions in PEC-SIM:CS:CMC (1:2:1) produced significant improvements (*p* < 0.05) at 15 and 60 min compared to SIM-RM. The expansion of the CS interpolymeric chains and the increase in intramolecular hydrogen bonds promote hydration and improve the SIM dissolution rate. Finally, the first-order and Korsmeyer–Peppas models were the best fitting models for PEC-SIM:CS:CMC (1:2:1) because of its fast release kinetics. These polyelectrolyte complexes with a large number of free protonated amino groups (NH_3_^+^) have great potential to improve the dissolution profiles of poorly soluble drugs. These polyelectrolyte complexes with high CS ratios could produce similar decreases in the crystallinity of other poorly soluble drugs and increase their wettability on the network surface due to their ionic interactions with the dissolution medium, resulting in improved drug dissolution.

## Figures and Tables

**Table 1:** Composition of polyelectrolyte complexes, type of interaction (FTIR), dissolution studies and adjustments to different kinetic studies.

CS: Anionic Polymer Drug	FTIR (Interaction Type)	Dissolution Study	Kinetic Study	Reference
**CS:CMC (1.5:1)** **5-Fluorulacilo (5-FU)**	5-FU/CMC interactions CS/CMC interactions Hydrogen bonds	SGF pH 2.0 and SIF (pH 6.8; pH 7.4) -pH 1.2 < 20% -pH 6.8 ~ 60% 2–3 h	First-order Kinetic at pH 6.8	[14]
**CS:Alginate (2:1)** **Cisplatin**	Non interactions were observed	Phosphate buffer pH 7.4 ~60% 2 h	First-order Kinetic	[13]
**CS:CMC (3:1)** **Vancomycin**	CS/CMC interactions	Acid buffer pH 2.0 30% 2 h	Pseudo-zero-order	[15]
**CS:CMC (1:1)** **CS:CMC (1:9)** **Chlorhexidine (CLX)**	CLX/CMC interactions CS/CMC interactions	Phosphate buffer pH 4.5 CS:CMC (1.1) ~ 70% 1 h CS:CMC (0.1:0.9) < 20% 6 h	Non-kinetic fit	[16]
**CS:Xanthan (1:1)** **Ibuprofen**	CS/Xanthan interaction Hydrogen bonds	Phosphate buffer pH 7.2 >90% 60 min	Modified Fickian diffusion mechanism *n* = 0.406; *r^2^* = 0.9697	[8]
**CS: Alginate (0.1:0.1)** **Lovastatin (LOV)**	LOV/complex interactions CS/CMC interactions Hydrogen bonds	SIF (pH 7.4) >60% 3 h	Fast stage Fickian diffusion (*n* < 0.38;*r^2^* > 0.9825)	[2]
**CS: Alginate (1:2)** **Lovastatin (LOV)**	LOV/complex interactions CS/CMC interactions Hydrogen bonds	SIF (pH 4.5; 6.5; 7.4) pH 7.4 >60% 3 h	pH 7.4 zero-order kinetics (*r^2^* = 0.985) pH 6.5 first-order kinetics (*r^2^* = 0.987) pH 4.5 first-order kinetics (*r^2^* = 0.989)	[3]
**CS:CMC (1:1)** **Atorvastatin (AT)**	AT/complex interactions CS/CMC interactions Hydrogen bonds	SIF pH 5.0 and pH 7.0 pH 5.0 >15% 4 h pH 7.0 >25% 4 h	Burst effect. Non-kinetic fit at both pH 5.0 and pH 7.0	[1]
**CS:Xanthan (1:1)** **Rosuvastatin (ROS)**	CS/Xanthan low interactions	Phosphate buffer pH 7.4 >60% 2 h	Non-kinetic fit	[12]
**CS:CMC (2:2)** **Congo red (CR)** **Methylene blue (MB)**	CS/CMC interactions Hydrogen bonds	Phosphate buffer pH 4.0 >60% 1 h	CR Pseudo-second-order (*r^2^* > 0.9620–0.9881) MB Pseudo-first-order (*r^2^* > 0.9620–0.9881)	[17]

**Table 2:** Zero-order, first-order, Higuchi, and Korsmeyer–Peppas kinetic models applied for SIM release from SIM-RM, the physical mixture PM-SIM:CS:CMC (1:1:2) and the three polyelectrolyte complexes (PEC-SIM:CS:CMC (1:1:1), PEC-SIM:CS:CMC (1:1:2) and PEC-SIM:CS:CMC (1:2:1)) with corresponding correlation coefficient (*r*^2^), release rate constant of zero-order *K*_0_ (min^−1^), first-order K_1_ (min^−1^), Higuchi K*_H_* (min^−1/2^) and Korsmeyer–Peppas K*_d_* (min^−*n*^).

Formulations	Kinetic Models	K	*r^2^*
SIM-RM	Zero-order	0.4361	0.9842
	First-order	−0.0076	0.9980
	Higuchi model	5.9106	0.9944
PM-SIM:CS:CMC (1:1:2)	Zero-order	0.7725	0.9799
	First-order	−0.0094	0.9656
	Higuchi model	7.1215	0.9761
PEC-SIM:CS:CMC (1:1:1)	Zero-order	1.5143	0.8948
	First-order	−0.0300	0.9686
	Higuchi model	14.165	0.9655
PEC-SIM:CS:CMC (1:1:2)	Zero-order	1.3013	0.9832
	First-order	−0.0145	0.9803
	Higuchi model	12.276	0.9835
PEC-SIM:CS:CMC (1:2:1)	Zero-order	1.6181	0.9637
	First-order	−0.0400	0.9976
	Higuchi model	12.766	0.9950

**Table 3:** Korsmeyer–Peppas kinetic model applied for SIM release from SIM-RM, the physical mixture PM-SIM:CS:CMC (1:1:2) and the three polyelectrolyte complexes (PEC-SIM:CS:CMC (1:1:1), PEC-SIM:CS:CMC (1:1:2) and PEC-SIM:CS:CMC (1:2:1)) with corresponding correlation coefficient (*r*^2^), release rate constant of Korsmeyer–Peppas *K_d_* (min*^−n^*) and diffusion exponent (*n*).

Formulations	Korsmeyer–Peppas Kinetic Model		
	*n*	*K_d_*	*r^2^*
SIM-RM	0.4597	0.8284	0.9955
PM-SIM:CS:CMC (1:1:2)	1.0395	0.3344	0.9712
PEC-SIM:CS:CMC (1:1:1)	0.8584	0.4834	0.9384
PEC-SIM:CS:CMC (1:1:2)	0.5921	0.5912	0.9153
PEC-SIM:CS:CMC (1:2:1)	0.4023	1.3057	0.9860

## Data Availability

Not applicable.
